# Detection of nuclei in 4D Nomarski DIC microscope images of early Caenorhabditis elegans embryos using local image entropy and object tracking

**DOI:** 10.1186/1471-2105-6-125

**Published:** 2005-05-24

**Authors:** Shugo Hamahashi, Shuichi Onami, Hiroaki Kitano

**Affiliations:** 1 Kitano Symbiotic Systems Project, ERATO, Japan Science and Technology Corporation, M31 6A, 6-31-15 Jingumae, Shibuya, Tokyo 150-0001, Japan; 2 Graduate School of Science and Technology, Keio University, 3-14-1 Hiyoshi, Kohoku, Yokohama 223-8522, Japan; 3 Institute for Bioinformatics Research and Development (BIRD), Japan Science and Technology Agency, 5-3 Yonbancho, Chiyoda, Tokyo 102-0081, Japan; 4 Sony Computer Science Laboratories, Inc., 3-14-13 Higashi-Gotanda, Shinagawa, Tokyo 141-0022, Japan

## Abstract

**Background:**

The ability to detect nuclei in embryos is essential for studying the development of multicellular organisms. A system of automated nuclear detection has already been tested on a set of four-dimensional (4D) Nomarski differential interference contrast (DIC) microscope images of *Caenorhabditis elegans *embryos. However, the system needed laborious hand-tuning of its parameters every time a new image set was used. It could not detect nuclei in the process of cell division, and could detect nuclei only from the two- to eight-cell stages.

**Results:**

We developed a system that automates the detection of nuclei in a set of 4D DIC microscope images of *C. elegans *embryos. Local image entropy is used to produce regions of the images that have the image texture of the nucleus. From these regions, those that actually detect nuclei are manually selected at the first and last time points of the image set, and an object-tracking algorithm then selects regions that detect nuclei in between the first and last time points. The use of local image entropy makes the system applicable to multiple image sets without the need to change its parameter values. The use of an object-tracking algorithm enables the system to detect nuclei in the process of cell division. The system detected nuclei with high sensitivity and specificity from the one- to 24-cell stages.

**Conclusion:**

A combination of local image entropy and an object-tracking algorithm enabled highly objective and productive detection of nuclei in a set of 4D DIC microscope images of *C. elegans *embryos. The system will facilitate genomic and computational analyses of *C. elegans *embryos.

## Background

The position of the nucleus is a crucial piece of information in any study of the development of multicellular organisms. A fertilized egg – a single cell – develops into a multicellular organism through many spatially and temporally dynamic cellular activities, including cell division, cell migration, cell differentiation, cell fusion, and cell death. Often, these dynamic cellular activities are described in terms of the positions of the nuclei, and the roles and mechanisms of those cellular activities are studied using these descriptions of cellular activities because the nucleus is generally positioned at the center of a cell and is the most noticeable organelle in a cell [1]. The position of the nucleus is usually identified from images captured through a microscope. Therefore, detection of the nucleus in microscope images is essential for studying the development of multicellular organisms.

The nucleus is usually detected manually on these microscope images. However, manual detection reduces the objectivity and productivity of identification of nuclear position. The objectivity and productivity of such measurements are becoming critical in modern biology, where the importance of bioinformatics, computational biology, and genomics is increasing. High objectivity of measurements is strongly expected in bioinformatics and computational biology. In the large-scale data analyses typical of bioinformatics, the quality of the analysis depends largely on that of the data analyzed [2]. In the simulation analyses typical of computational biology, the decision-making step is a comparison between the simulation and *in vivo *measurement [3]. High productivity of measurements is strongly expected in genomics. Organisms have thousands of genes [4,5], and systematic study of the functions of all of these genes – a typical strategy in genomics – needs thousands of measurements [6].

The soil nematode *Caenorhabditis elegans *is the simplest multicellular organism that has been most extensively studied in biology [7,8]. Because of the simplicity of this organism, results from its study constitute a foundation for our understanding of higher multicellular organisms. In *C. elegans*, the position of the nucleus is usually identified from images obtained through a Nomarski differential interference contrast light microscope, hereafter called a *DIC microscope *[9]. Three-dimensional (3D) positions of the nuclei are identified from a set of images recorded in multiple focal planes, and time-dependent changes in these positions are followed in a set of images recorded in multiple focal planes and at multiple time points. The 4D DIC microscope is an automated system that records DIC microscope images in multiple focal planes and at multiple time-points [10,11]. To help follow time-dependent changes in the 3D positions of nuclei in a set of images recorded by the 4D DIC microscope system (hereafter called a set of *4D DIC microscope images*), two computer-assisted systems have been developed, namely SIMI BioCell [12] and 3D-DIASemb [13]. SIMI BioCell is a graphical user interface that displays a set of 4D DIC microscope images, helps to identify the positions of nuclei, and records these identified positions. 3D-DIASemb is similar to SIMI BioCell but can also record and display the perimeter of the nucleus and cell. Although both of these systems help greatly to follow time-dependent changes in the 3D positions of nuclei, the nuclei are still detected manually and nuclear detection is therefore still a laborious task. As a result, the objectivity and productivity of identification of nuclear positions are still low.

Automation of nuclear detection increases the objectivity and productivity of identification of nuclear positions. Yasuda *et al*. [14] attempted to automate nuclear detection by using several edge detection operators [15,16]. Their automated system detected nuclei from the two- to eight-cell stages in a specific set of 4D DIC microscope images. However, their system required laborious hand-tuning of parameters every time a new set of 4D DIC microscope images was applied, because the edge detection operators were very sensitive to differences in image quality (e.g., brightness, contrast) among sets of images; the differences could be controlled but not eliminated. In addition, their system could not detect nuclei that were in the process of cell division, because detection of nuclei relied on the nucleus being round (and therefore not in the process of division). Unless the positions of the dividing nuclei are known, it is difficult to follow the cell division pattern of embryos. Therefore, the system of Yasuda *et al*. [14] requires marked improvement before it can be used in research.

We developed a system that automates the detection of nuclei in *C. elegans *embryos. Our system uses local image entropy [17] and an object-tracking algorithm [18-20] to automate the detection of nuclei in sets of 4D DIC microscope images. Because local image entropy is not sensitive to differences in image quality among sets of images, our system can be applied to different sets without the need to change the system parameters. Because the object-tracking algorithm is independent of the process of cell division, our system detects nuclei both in and not in the process of cell division. Here, we show that our system can effectively detect nuclei in a *C. elegans *embryo from fertilization to the onset of gastrulation, i.e., from the one- to 24-cell stages.

## Results

### Appearance of nuclei in images obtained by the 4D DIC microscope system

**Figure 1:**
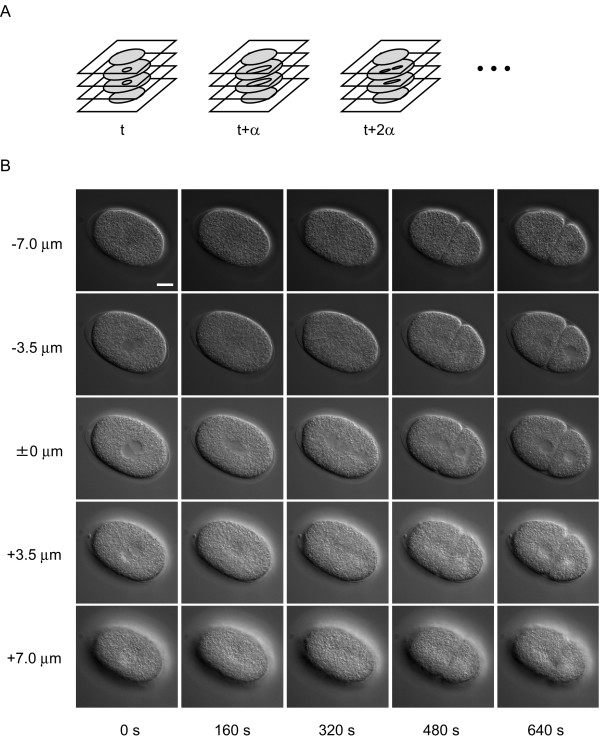
**Overview of 4D DIC microscope images of *C. elegans *embryo**. (A) Schematic of 4D DIC microscope images. Digital images of a developing embryo were recorded in multiple focal planes and a set of multifocal images was recorded with a fixed time interval, α. (B) Example of 4D DIC microscope images of a *C. elegans *embryo. Each column shows multifocal images recorded at a specific time point, with 3.5 μm between two focal planes. Each row shows time-lapse images recorded in a specific focal plane with 160 s between two time points. Bar is 10 μm.

The appearance of the nuclei of *C. elegans *embryos in 4D DIC microscope images (Figure 1A, B) varies among different focus levels and different developmental stages. The nucleus appears as a smooth, round region in the center of the cell, the cytoplasm of which appears as a rough region at all developmental stages. The boundary of the nucleus is apparent when the focus level is close to the level of the center of the nucleus (0 μm, 0 s in Figure 1B). As the focus level becomes higher or lower, the nucleus becomes smaller, reflecting the 3D shape of the nucleus, and the boundary of the nucleus becomes blurred (-3.5 μm and +3.5 μm in Figure 1B). The nucleus becomes invisible when the focus level goes beyond the level of the upper or lower end of the nucleus (-7.0 μm and +7.0 μm in Figure 1B). As the embryo develops, the number of cells in the embryo increases through repeated cell divisions, each of which produces two daughter cells from a single mother cell. When cell division begins, the nucleus begins to elongate and the boundary of the nucleus becomes blurred (160 s in Figure 1B). As cell division progresses, the nucleus continues to elongate (320 s in Figure 1B). The elongated nucleus is fragmented into several pieces (480 s in Figure 1B), which then form daughter nuclei in two daughter cells (640 s in Figure 1B). The size of the nuclei gradually decreases as the embryo develops and the number of nuclei increases (8 μm in diameter at the one-cell stage and 5 μm at the 24-cell stage). Although the appearance of the nuclei in the images varies among different focal planes and different developmental stages, a smooth image texture is a common feature of the appearance of nuclei. Our image-processing algorithm uses this feature to detect nuclei in the images (see next section).

### Detection of nuclei using regions of low local image entropy

To detect nuclei in the 4D DIC microscope images, we used a common feature of nuclei in the images, that is, their smooth image texture (see previous section, Figure 1B). To quantify the smoothness of image texture in various regions of an image, we used local image entropy [17], which computes the image entropy [21] of a small area surrounding a point of interest in an image. Image entropy represents the smoothness of image texture; its value becomes high when the texture is rough and low when the texture is smooth. Because smooth image texture is a common feature of the appearance of nuclei in 4D DIC microscope images, we expected local image entropy to be lower in the nuclei than in the cytoplasm. An important feature of image entropy is low sensitivity to differences in image quality, particularly in terms of the brightness of the image. Therefore, we expected that local image entropy would quantify the smoothness of image texture in multiple images in a manner that was not sensitive to differences in quality among images.

We defined an image conversion using local image entropy as follows. Let [*x*_*ij*_] be the matrix representing a digitized input image. Then the result of image conversion using local image entropy in an *X *× *Y *pixel window is an image [*y*_*ij*_], where the value of *y*_*ij *_equals the entropy of the input image lying in the *X *× *Y *pixel window *W*_*ij *_whose top left is pixel *x*_*ij*_. The image entropy is , where *N *is the number of gray levels and *P*(*k*) is the probability of occurrence of gray level *k *in window *W*_*ij*_. Because of the presence of the window, the number of columns and rows of [*y*_*ij*_] is smaller than those of [*x*_*ij*_] by *X *- 1 and *Y *- 1, respectively.

**Figure 2:**
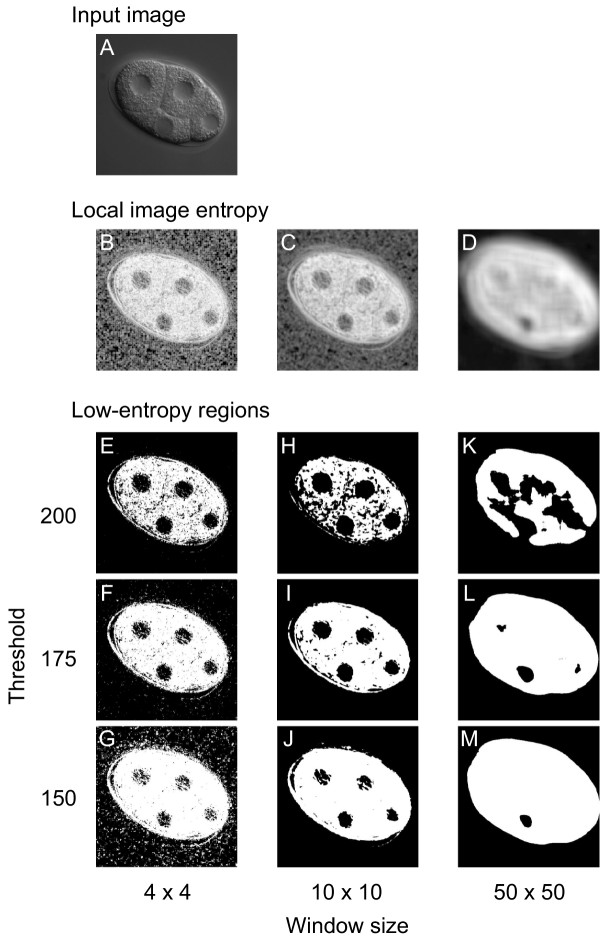
**Effect of window size and threshold value on production of low-entropy regions**. (A) Input image. Low-entropy regions were produced from an image of a four-cell-stage embryo using various window sizes and threshold values. (B–D) Effect of window size on image conversion using local image entropy. The input image was applied to the image conversion using window sizes of 4 × 4 (B), 10 × 10 (C), and 50 × 50 (D) pixels. Darker colors represent lower local image entropies. (E–M) Effect of window size and threshold value on low-entropy regions. Low-entropy regions (black) were produced using threshold values of 200 (E, H, K), 175 (F, I, L), or 150 (G, J, M) from the images resulting from the image conversion, using window sizes of 4 × 4 (E–G), 10 × 10 (H–J) or 50 × 50 (K–M) pixels. A window of 10 × 10 pixels corresponds to that of 1 μm × 1 μm.

To determine whether local image entropy could effectively distinguish nuclei from cytoplasm in 4D DIC microscope images, we converted the images using various window sizes (from 2 × 2 to 50 × 50 pixels, results for 4 × 4, 10 × 10 and 50 × 50 pixels are shown in Figure 2). As expected, local image entropy was lower (darker) in the nuclei than in the cytoplasm (e.g., 10 × 10 window size in Figure 2). When we used a large (50 × 50) window, the difference in local image entropy between nuclei and cytoplasm became smaller. When we used a small (4 × 4) window, high-entropy spots (bright spots) appeared throughout the images. These results indicate that local image entropy effectively distinguishes nuclei from cytoplasm in 4D DIC microscope images. For our images, 10 × 10 pixels (1 μm × 1 μm) appeared likely to be the optimal size of the window. We investigated 25 widely-used texture measures selected from all four texture analysis methods categorized by Tuceryan and Jain [22] and confirmed that local image entropy provides the best performance among those texture measures to distinguish between nuclei and cytoplasm (see Additional file 1).

To detect nuclei using this difference in local image entropy between nuclei and cytoplasm, we applied thresholding [23] to the images resulting from the image conversion and produced *low-entropy regions *(Figure 2E–M). The low-entropy regions were produced as follows: neighboring pixels whose local image entropy was lower than the threshold were grouped, and the resulting group was defined as a low-entropy region. As expected, many of these low-entropy regions corresponded to nuclei in the original images, whereas the size and number of the regions depended on the threshold value. The shapes of the low-entropy regions approximated those of corresponding nuclei when the threshold value was set to 175 (Figure 2F, I, L). As the threshold value decreased, the regions became smaller and more fragmented (Figure 2G, J, M). As the threshold value increased, the regions became larger and more aggregated (Figure 2E, H, K). These results indicate that low-entropy regions can be used to detect nuclei in 4D DIC microscope images. For our images, 175 was likely to be the optimal threshold value. In addition to the low-entropy regions that corresponded to nuclei, many low-entropy regions were produced that did not correspond to nuclei. These low-entropy regions corresponded to regions that have similar (smooth) image textures to that of the nucleus, such as the boundaries between cells and the spaces between the embryo and the eggshell.

### Nuclear detection using low-entropy regions

**Figure 3:**
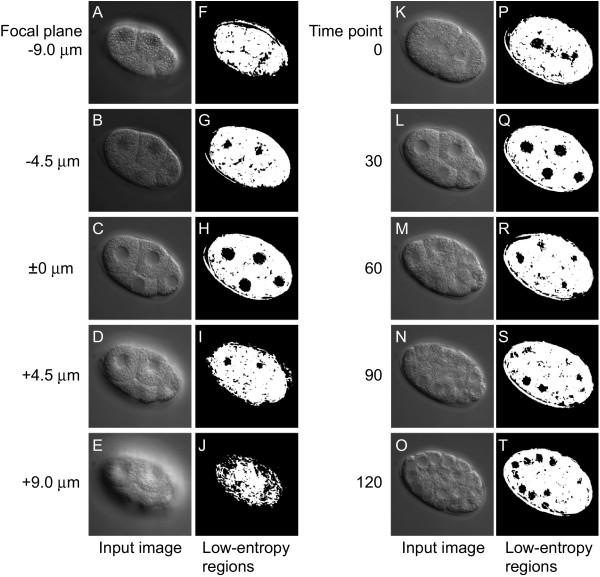
**Low-entropy regions of different focal planes and different time points**. (A – E and K–O) Input images. (F–J and P–T) Low-entropy regions (black) produced from the input images. Low-entropy regions were produced from multifocal images of an embryo at a specific time point in the four-cell stage with 4.5 μm between two focal planes (A–E) and from time-lapse images of an embryo at a specific focal plane with 30 min between two time points (K–L). The low-entropy regions produced are displayed to the right of each input image. The window size was 10 × 10 pixels and the threshold value was 175.

We evaluated the performance of nuclear detection in a set of 4D DIC microscope images by using low-entropy regions. For the evaluation, we produced low-entropy regions from five sets of images of *C. elegans *embryos using a 10 × 10 pixel window and a threshold value of 175 (Figure 3). Each set of images consisted of 10,080 images (56 focal planes × 180 time points = 10,080 images). We then calculated the sensitivity and specificity as measures of performance.

Sensitivity was defined as the ratio of the sum of the number of nuclei detected at each time point to the sum of the number of nuclei existing at each time point. A nucleus was considered to be "detected" at a specific time point when it was detected by at least one low-entropy region at any focal plane at this specific time point. This definition of sensitivity is reasonable because of the difficulty in specifying the number of low-entropy regions that are expected to detect a given nucleus. The following three factors underlie this difficulty. First, a single nucleus is usually detected by several low-entropy regions in different focal planes at a single time point. Second, a single nucleus is sometimes detected by several low-entropy regions in the same focal plane at a single time point. Third, it is difficult to determine which focal plane is the top end and which is the bottom end of the focal planes at which a given nucleus is expected to be detected in low-entropy regions, because the appearance of the nucleus becomes gradually blurred as the focal plane becomes farther from the center of the nucleus (Figure 1B).

Specificity was defined as the ratio of the number of low-entropy regions detecting nuclei to the number of low-entropy regions produced. Because local image entropy is not sensitive to differences in image quality, particularly in terms of the brightness of the image, we expected that the performance of nuclear detection by examination of low-entropy regions would differ little among sets of 4D DIC microscope images.

We obtained perfect (= 1.0) sensitivity for all sets of images from the one- to the 24-cell stages (Table 1). All nuclei were detected at any time point independently of whether or not they were in the process of cell division. To confirm that this perfect sensitivity was not solely a feature of the five sets of images examined, we produced low-entropy regions from 44 sets of images of *C. elegans *embryos using 10 × 10 pixel windows and threshold values of 175, and then calculated the sensitivity. We obtained perfect sensitivity for all 44 sets of images of embryos from the one- to the 24-cell stages (data not shown). Sensitivity became imperfect in the later stages of embryogenesis, i.e., around the 44-cell stage or later (data not shown). In contrast, very low (< 0.10) specificity was obtained for all sets of images (Table 1). In summary, low-entropy regions could be used to detect nuclei in a set of 4D DIC microscope images of *C. elegans *embryos from the one- to 24-cell stages with very high sensitivity and very low specificity. The performance of nuclear detection by low-entropy regions differed little among sets of images.

### Selection of low-entropy regions using object-tracking algorithm in the forward direction of time

The very high sensitivity and very low specificity of nuclear detection by using low-entropy regions motivated us to develop a process that selected low-entropy regions that actually detected nuclei. To develop this process, we used spatial and temporal information on the nucleus. In terms of spatial information, we expected the nucleus to be detected by several low-entropy regions, each of which would overlap with another region in an adjacent focal plane at the same time point, because the radius of the nucleus (> 2.5 μm) was much larger than the distance between two adjacent focal planes (0.5 μm). Therefore, a low-entropy region would be more likely to detect a nucleus than others when it overlapped with a region that detected the nucleus in an adjacent focal plane at the same time point. In terms of temporal information, we expected the nucleus to be detected by several low-entropy regions, each of which would overlap with another region in the same focal plane at an adjacent time point, because the nucleus rarely moves more than a distance equal to its diameter (> 5 μm) within the time equal to the interval between two adjacent time points (40 s). Therefore, a low-entropy region would be more likely to detect a nucleus than others when the region overlapped with a region that detected the nucleus in the same focal plane at an adjacent time point.

**Figure 4:**
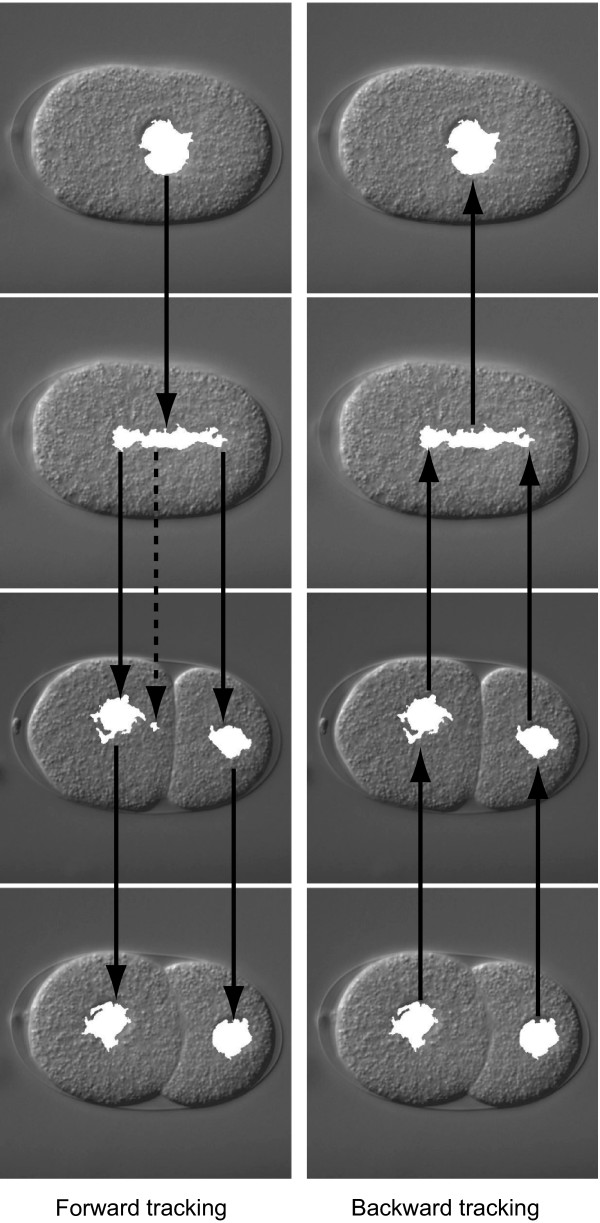
**Overview of forward and backward trackings**. Low-entropy regions selected by forward tracking are shown in the left column and those selected by backward tracking after the selection by forward tracking are shown in the right column. These low-entropy regions (white) are overlaid on their corresponding input images. Solid arrows represent the tracking of low-entropy regions. The broken arrow represents a dead-end branch of tracking.

To select low-entropy regions by using this spatial and temporal information, we used an object-tracking algorithm [18-20] (Figure 4). The tracking algorithm was composed of the following two recursive processes. First, a low-entropy region in focal plane *f *at time point *t *is selected if the region overlaps with a region that has been selected in either focal plane *f *- 1 or *f *+ 1 at time point *t*. Second, a low-entropy region at focal plane *f *at time point *t *is selected if the region overlaps with a region that has been selected in focal plane *f *at time point *t *- 1. Manual selection of a low-entropy region at time point 0 triggers these processes. We call this algorithm *forward tracking *because it tracks nuclei in the forward direction of time.

To examine whether forward tracking effectively selects low-entropy regions that can actually detect nuclei, we applied this algorithm to the low-entropy regions produced from five sets of 4D DIC microscope images of *C. elegans *embryos from the one- to 24-cell stages (Table 1). As expected, we obtained perfect sensitivity for nuclear detection by the selected low-entropy regions. All nuclei were detected at any time point, independently of whether or not they were in the process of cell division. Specificity was about 6.7 times better than before selection, although it was still far from perfect. These results indicate that forward tracking effectively selects low-entropy regions that can actually detect nuclei.

### Further selection of low-entropy regions using object-tracking algorithm in the backward direction of time

To further select low-entropy regions, we used another tracking algorithm. This algorithm, called *backward tracking*, used the same recursive processes as forward tracking, with the exception of the direction of tracking, i.e., it tracked nuclei in the backward direction of time (Figure 4). We expected that this backward tracking would be effective for selecting low-entropy regions after forward tracking, because forward tracking usually creates many dead-end branches (Figure 4), which consist of low-entropy regions that do not detect nuclei. Backward tracking selected low-entropy regions that were not included in these dead-end branches (Figure 4).

Backward tracking was composed of the following two recursive processes. First, a low-entropy region in focal plane *f *at time point *t *is selected if the region overlaps with a region that has been selected in either focal plane *f *- 1 or *f *+ 1 at time point *t*. Second, a low-entropy region in focal plane *f *at time point *t *is selected if the region overlaps with a region that has been selected in focal plane *f *at time point *t *+ 1. Manual selection of low-entropy regions at the last time point triggers the processes.

To examine whether backward tracking is effective for selection of low-entropy regions after forward tracking, we applied backward tracking to the five sets of low-entropy regions selected by forward tracking (Table 1). Again, we obtained perfect sensitivity for nuclear detection by low-entropy regions selected by backward tracking. All nuclei were detected at any time point independently of whether or not they were in the process of cell division. Sensitivity was markedly better than before backward tracking, although it was still far from perfect. These results indicate that backward tracking is effective for selection of low-entropy regions after forward tracking.

### Excellent selection of low-entropy regions using object-tracking algorithm, depending on the extent of overlap between two regions

The very high sensitivity but far lower perfect specificity (0.56 in average) of low-entropy regions selected by the combination of forward and backward trackings motivated us to develop a process that would more effectively select low-entropy regions that could detect nuclei. To develop this process, we used more detailed spatial and temporal information on the nucleus. In terms of more detailed spatial information, we expected the nucleus to be detected by several low-entropy regions, each of which overlapped to a large extent with one of the others in an adjacent focal plane at the same time point, because the 3D shape of the nucleus is usually simple. Therefore, a low-entropy region would become more likely to detect a nucleus when the region overlapped to a large extent with a region that detected the nucleus in an adjacent focal plane at the same time point. In terms of more detailed temporal information, we expected that a nucleus would be detected by several low-entropy regions, each of which overlapped to a certain extent with another in the same focal plane at two adjacent time points, because the nucleus usually moves much less than a distance equal to its diameter within the time equal to the interval between two adjacent time points. Therefore, a low-entropy region would become more likely to detect a nucleus when the region overlapped with a region that detected the nucleus in the same focal plane at two adjacent time points, and when both regions overlapped by a large extent.

To select low-entropy regions using this more detailed spatial and temporal information, we introduced a *minimum overlap ratio *to the forward and backward trackings. The minimum overlap ratio between two low-entropy regions was defined as the smallest ratio of the number of pixels shared by these two regions to the number of pixels making up each region. Thus, when the minimum overlap ratio between two overlapping regions increases, the two regions overlap to a greater extent, i.e., the two regions are more likely to detect the same nucleus. In the forward and backward trackings, we used this minimum overlap ratio to select pairs of low-entropy regions that overlapped to an extent greater than a prefixed value – i.e., pairs of low-entropy regions that were more likely to detect the same nucleus than a prefixed likelihood.

Forward tracking with a minimum overlap ratio was composed of the following two recursive processes. First, a low-entropy region in focal plane *f *at time point *t *is selected if the region overlaps with a region that has been selected either at focal plane *f *- 1 or *f *+ 1 at time point *t *by a minimum overlap ratio more than the threshold *T*_*f*_. Second, a low-entropy region in focal plane *f *at time point *t *is selected if the region overlaps with a region that has been selected in focal plane *f *at time point *t *- 1 by a minimum overlap ratio more than the threshold *T*_*t*_. Manual selection of a low-entropy region at time point 0 triggers the processes in the same way as with the original forward tracking.

Backward tracking with a minimum overlap ratio is composed of the same recursive processes as forward tracking with a minimum overlap ratio, except that the direction of tracking is reversed – i.e., it tracks nuclei in the backward direction of time in the same way as with the original backward tracking. Manual selection of low-entropy regions at the last time point triggers the processes in the same way as with the original backward tracking. We expected that, as *T*_*f *_and *T*_*t *_increased, the selected low-entropy regions would become more likely to detect nuclei.

To examine whether the combination of forward and backward trackings with minimum overlap ratio (hereafter called *advanced forward and backward trackings*) would more effectively select low entropy regions than a combination of the original forward and backward trackings, we applied this combination of advanced forward and backward trackings to the low-entropy regions produced from five sets of 4D DIC microscope images of *C. elegans *embryos from the one- to 24-cell stages. Various sets of *T*_*f *_and *T*_*t *_were examined (Tables 2, 3). As expected, as *T*_*f *_and *T*_*t *_increased, the specificity of detection by the selected low-entropy region increased, whereas the sensitivity of detection by the region decreased. We found many sets of *T*_*f *_and *T*_*t *_that provided very high specificity (= 1.0), and several of them also provided perfect sensitivity (for example, *T*_*f *_= 70% and *T*_*t *_= 4% in Table 3). In this set of *T*_*f *_and *T*_*t*_, the selected low-entropy regions nearly perfectly detected all nuclei at any time point, independently of whether or not the nuclei were in the process of cell division. These results indicate that the combination of advanced forward and backward trackings more effectively selected low-entropy regions than did the combination of original forward and backward trackings. When an optimal set of *T*_*f *_and *T*_*t *_was applied, the combination of advanced forward and backward trackings nearly perfectly selected low-entropy regions that could detect nuclei.

## Discussion

We developed a system that automates the detection of nuclei in a set of 4D DIC microscope images of *C. elegans *embryos. One major advantage of this system is the use of local image entropy to quantify the appearance of the nucleus in the images. Our previous system used edge detection operators to quantify the appearance of the nucleus [14]. Because these operators were sensitive to differences in image quality (e.g., brightness, contrast) among sets of images, the previous system required laborious hand-tuning of system parameters each time a new image set was used (see Additional file 1). Local image entropy is not sensitive to differences in image quality among sets of images because it represents the smoothness of the image texture (see Additional file 1). Therefore, our system can be applied to different image sets without the need to change the system parameters. We applied five sets of 4D DIC microscope images to our system, and the system detected the nuclei in these sets with similar sensitivity and specificity when we used the same parameter values (Table 1). This reduced sensitivity to differences in image quality makes our system applicable to research. We can apply this system to sets of 4D DIC microscope images of mutant *C. elegans *embryos (see Additional file 2) and embryos in which specific genes are silenced by RNA interference (see Additional file 3).

Another major advantage of our system is the use of object-tracking algorithms to examine all regions with the features of the image texture of the nucleus (i.e., low local image entropy) in a set of 4D DIC microscope images and to select regions that can actually detect nuclei. A DIC image of a *C. elegans *embryo contains many regions that have similar (smooth) image textures to that of the nucleus but that do not actually correspond to the nucleus, such as the boundaries between cells and the spaces between the embryo and the eggshell (Figure 3). Thus, in addition to image texture, other features of the nucleus are needed to completely distinguish the nucleus. Our previous system used the (round) shape of the nucleus that was not in the process of cell division in addition to the feature of image texture, as quantified by edge detection operators [14]. This previous system could not detect nuclei in the process of cell division. The object-tracking algorithm in our new system uses spatial and temporal information on the nucleus, and this information is independent of the process of cell division. Thus, our system detects all nuclei – whether or not the cell is dividing – at every time point from one- to 24-cell stages. This continuous detection of nuclei is a great help in following the cell division pattern of the embryo.

Our system effectively detected nuclei over a markedly longer developmental period than did the previous system, i.e., from the one- to 24-cell (Tables 2, 3) stages compared with only the two- to eight-cell stages [14]. This extension of the period of effective nuclear detection primarily results from the very high sensitivity of nuclear detection by low-entropy regions before forward and backward trackings (Table 1). The sensitivity and specificity of nuclear detection by these "original" low-entropy regions depend on the parameters used to produce the regions (i.e., window size and entropy threshold): the higher the sensitivity, the lower the specificity. Our system uses a set of values for these parameters that provides very high sensitivity and very low specificity of nuclear detection by the original low-entropy regions (Table 1), because subsequent forward and backward trackings effectively distinguish those regions that actually detect nuclei from those that do not.

The previous system used a two-step strategy similar to ours: i.e., regions that had the image texture of the nucleus were produced using edge detection operators, and from these "likely nuclear" regions, those that actually detected nuclei were selected using the shape of the nucleus. The sensitivity and specificity of nuclear detection by these "original" likely nuclear regions depended on the parameters used to produce the regions. However, the shape-dependent selection of likely nuclear regions was far less effective than the selection of low-entropy regions by forward and backward trackings. Thus, the previous system used a set of parameter values that provided markedly lower sensitivity and markedly higher specificity of nuclear detection by the original likely nuclear regions than by the original low-entropy regions. In the current study, we found very high sensitivity of nuclear detection by the original low-entropy regions up to the 44-cell stage (data not shown). Thus, improvement in the selection of low-entropy regions will further extend the period of effective nuclear detection. We are developing an improved system that uses both a tracking algorithm and the known shape and size of nuclei in non-dividing cells to select low-entropy regions.

Fluorescent labeling of nuclei is a method that has recently been developed for identifying the positions of the nuclei in living *C. elegans *embryos [24,25]. With this method, the genetic information of an embryo is artificially modified so that the embryo expresses nuclear protein fused with fluorescent protein, such as histone H2B fused with green fluorescent protein (GFP) [25]; the embryo is illuminated by excitatory light (e.g., blue or UV light for GFP), and the expressed fusion protein produces light of a specific color (e.g., green for GFP). Because the nuclei are labeled with a specific color, detection of the nuclei is much easier than that using the DIC microscope. However, the development of the embryo expressing the fusion protein may differ from that of the intact embryo because of the presence of GFP or the modification of genetic information [26-28]. Fluorescent labeling can be used to visualize nuclei for a markedly shorter period than with the DIC microscope because of photobleaching: i.e., the intensity of fluorescence of the fusion protein decreases because of exposure of the protein to the excitatory light [29], although the amount of photobleaching can be reduced by the use of multiphoton fluorescence imaging [30]. In contrast, the DIC microscope can be used to visualize the nuclei of an intact embryo throughout the development of *C. elegans*. Therefore, to describe the precise position of nuclei in living *C. elegans *embryos, identification of the position of the nucleus using the DIC microscope seems more suitable than that using fluorescent labeling of nuclei.

A major drawback of our system is the need for manual selection of low-entropy regions at the first and last time points. These manual operations may reduce the objectivity and productivity of our system, because selection is determined by the operator. However, slight differences in manual selection at the first and the last time points does not influence the automated selection of low-entropy regions in between these points, because the automated selections select all regions that overlap with other selected regions in the adjacent focal plane at the same time point or in the same focal plane at the adjacent time point. Thus, usually our system objectively detects nuclei in between the first and last time points. Manual selection of low-entropy regions at the first and the last time points could still reduce the productivity of our system, because these manual selections usually take about 10 min. However, our system still markedly increases the productivity of identification of the positions of the nuclei in *C. elegans *embryos, because manual selection of low-entropy regions for all time points from the one- to 24-cell stages (56 focal planes × ~120 time points = ~6720 images) takes more than 50 h. Our system needs about 135 min for computation (120 min for the production of low-entropy regions and 15 min for the forward and backward trackings) and 10 min for manual operations to detect all the nuclei in a set of 4D DIC microscope images of a *C. elegans *embryo recorded from the one- to 24-cell stages. These times for computation and manual operations are acceptable in research. The selection of low-entropy regions at the first and last time points will be automated, most likely by using known properties of nuclei, such as the known shapes and sizes of nuclei in non-dividing cells.

The low-entropy regions before selection by the forward and backward trackings failed to detect nuclei at around the 44-cell stage or later. Because the window size (10 × 10 pixels) and the threshold value (175) used in this experiment appear likely to be optimal for our system, the result indicates that the limit of the nuclear detection system presented here is around the 44-cell stage. We believe that this limit comes from the reduction in size of the cells during embryogenesis. As the size of the cells decreases during embryogenesis, the distance between the nucleus and cell membrane decreases. Usually at around the 44-cell stage, some nuclei are positioned so close to the cortex of the embryo that a 10 × 10 pixel window cannot produce a high-entropy (> 175) boundary between the nucleus and the image background; the texture of the image background is smooth (Figure 2), and thus the local image entropy in the image background is as low as that in the nucleus. In this situation, the low-entropy regions corresponding to the cortically positioned nucleus merge with the low-entropy regions corresponding to the image background. Because our nuclear detection system removes the low-entropy regions corresponding to the image background, the low-entropy regions produced by our system fail to detect the cortically positioned nucleus. To overcome this limitation, modulation of the window size and/or the threshold value depending on the embryonic stage and/or position of the nucleus within the embryo (central or cortical) might be effective. We observed that low-entropy regions produced using a smaller (< 10 × 10 pixel) window size and/or smaller (< 175) threshold value successfully discriminated between such cortically positioned nuclei and the image background in the later stages of embryogenesis.

Our system is applicable to research programs that require high objectivity and/or productivity of identification of the positions of the nuclei in *C. elegans *embryos. Because the sensitivity and specificity of nuclear detection by our system depend on the thresholds for minimum overlap ratios (*T*_*f *_and *T*_*t*_), the values of these thresholds should be specified when the system is applied to a specific study. We often use *T*_*f *_= 70% and *T*_*t *_= 4%, because sensitivity is often more important than specificity in our research. We applied this system to our automated cell division pattern measurement system for *C. elegans *embryos; the measurement system was used in our large-scale cell division pattern analysis of gene-knockout *C. elegans *embryos [31]. The cell division pattern analysis will provide new opportunities for bioinformatics in studies of the development of multicellular organisms [32]. In addition, this system has been used to measure the positions of the male pronucleus (the sperm-derived nucleus) in a very early *C. elegans *embryo; the measurements were compared with computer simulations to determine the mechanism that specifies the positions of the male pronucleus during the very early period of *C. elegans *development [33]. To calculate the precise 3D shape and/or position of a nucleus from the low-entropy regions produced by this system, we need to consider the DIC shear angle, because the angle makes a substantial artifact in DIC images [34]. Because of its high objectivity and productivity of measurement, our system will contribute greatly to studies of the development of multicellular organisms.

## Conclusion

We have presented a system that automates the detection of nuclei in a set of 4D DIC microscope images of *C. elegans *embryos. The system can be applied to multiple image sets without the need to change parameter values. It can be used to detect nuclei that are in the process of cell division and can detect nuclei with very high sensitivity and specificity from fertilization to the onset of gastrulation, i.e., from the one- to 24-cell stages, enabling highly objective and productive identification of the positions of nuclei in *C. elegans *embryos. The system is applicable to comparisons between *in vivo *measurement and computer simulation and to systematic cell division pattern analysis of knockout embryos.

## Methods

### Preparation of 4D DIC microscope images of *C. elegans *embryos

The Bristol N2 *C. elegans *was cultured under standard conditions [35]. An embryo immediately after fertilization (before meeting of the female and male pronuclei) was dissected from a hermaphrodite and mounted on a 2% agar pad on a glass slide, covered with a coverslip, and sealed with petroleum jelly. Nomarski DIC images were obtained using a Leica DMRE microscope equipped with an HCX PL APO 100×/1.40 NA objective, whose illumination intensity and objective-side Wollaston prism were adjusted to obtain images of the same quality. Digital images of 600 × 600 pixels with 256 gray levels (0.1 μm per pixel) were recorded with an ORCA CCD Camera (Hamamatsu Photonics), and the recording system was controlled by IP Lab 3.5 software (Scanalytics). Digital images of the developing embryo were recorded at 22°C in 56 focal planes, with a distance of 0.5 μm between two focal planes, and a set of 56 focal plane images was recorded every 40 s for 2 h.

### Calculation of sensitivity and specificity of nuclear detection

Low-entropy regions that actually detected nuclei were manually selected in five sets of 4D DIC microscope images of an embryo. The resulting five sets of low-entropy regions were used as references to calculate the sensitivity and specificity of nuclear detection. Sensitivity was calculated using low-entropy regions of all time points from time point 0 to that corresponding to the end of the 24-cell stage. Specificity was calculated using low-entropy regions at 11 time points, obtained by sampling every 10 time points from the beginning of the two-cell stage to the end of the 24-cell stage. The number of time points from the beginning of the two-cell stage to the end of the 24-cell stage was 106 on average.

### Hardware and software environment

Because we needed to process many images, low-entropy regions were produced from sets of 4D DIC microscope images using a Beowulf-class PC cluster [36] consisting of 48 nodes, each of which used a 2-GHz Intel Pentium 4 processor, 1 GB of SDRAM memory, and a 100 Base-TX Ethernet card. Parallel Virtual Machine (PVM) software [37] was used for communications between the nodes. In our implementation, a single image in an image set was processed by a single CPU in the cluster. The forward and backward trackings for selection of low-entropy regions were processed on a single processor PC (2.2 GHz Intel Pentium 4 processor and 1 GB of RDRAM memory). The programs were written in C.

### Availability

Our software implementations are readily available on the web at .

## Authors' contributions

SH participated in conception, designed the system, tested the system, and drafted the manuscript. SO conceived of the study, contributed to the design, tested the system, and drafted the manuscript. HK participated in system design. All authors read and approved the final manuscript.

## Supplementary Material

Additional File 1 Detailed discussions about various measures of image texture. Click here for file

Additional File 2 **Figure 5 **Low-entropy regions in a *par-1 *embryo. Input images of a *par-1 *embryo are shown in the left column and low-entropy regions (black) are shown in the right column. Bar is 10 μm. Click here for file

Additional File 3 **Figure 6 **Low-entropy regions in a *tba-2 *(RNAi) embryo. Input images are shown in the left column and low-entropy regions (black) are shown in the right column. Bar is 10 μm. Click here for file

## Figures and Tables

**Table 1:** Performance of nuclear detection by low-entropy regions and those selected by forward and backward trackings

	set 1		set 2		set 3		set 4		set 5	
Low-entropy regions	0.06	(1.0)	0.08	(1.0)	0.06	(1.0)	0.08	(1.0)	0.08	(1.0)
Forward Tracking	0.38	(1.0)	0.48	(1.0)	0.55	(1.0)	0.44	(1.0)	0.53	(1.0)
Forward and backward trackings	0.44	(1.0)	0.55	(1.0)	0.67	(1.0)	0.50	(1.0)	0.63	(1.0)

Data are specificity and (sensitivity) for five sets of 4D DIC microscope images of a *C. elegans *embryo from the one- to 24-cell stages (1 set = 56 focal planes × ~120 time points = ~6720 images).

**Table 2:** Specificity of nuclear detection by low-entropy regions selected by advanced forward and backward trackings

	T_f_	T_t_											
		
		1 pixel^†^		4%		8%		12%		16%		20%	
Forward tracking	1 pixel^‡^	0.48	(0.06)	0.54	(0.11)	0.56	(0.11)	0.56	(0.10)	0.58	(0.10)	0.59	(0.10)
	30%	0.57	(0.08)	0.69	(0.11)	0.74	(0.11)	0.78	(0.10)	0.84	(0.09)	0.87	(0.07)
	50%	0.66	(0.11)	0.79	(0.11)	0.84	(0.08)	0.87	(0.07)	0.90	(0.06)	0.94	(0.03)
	70%	0.74	(0.12)	0.86	(0.08)	0.90	(0.07)	0.93	(0.05)	0.94	(0.05)	0.97	(0.03)
	90%	0.83	(0.10)	0.93	(0.04)	0.95	(0.03)	0.98*	(0.01*)	0.99*	(0.01*)	1.00*	(0.00*)

Forward and backward trackings	1 pixel^‡^	0.56	(0.08)	0.62	(0.13)	0.64	(0.13)	0.65	(0.12)	0.67	(0.13)	0.69	(0.13)
	30%	0.70	(0.10)	0.83	(0.12)	0.87	(0.09)	0.90	(0.07)	0.93	(0.05)	0.95	(0.03)
	50%	0.82	(0.12)	0.91	(0.07)	0.93	(0.05)	0.95	(0.05)	0.97	(0.04)	0.98	(0.02)
	70%	0.87	(0.11)	0.96	(0.05)	0.97	(0.04)	0.99	(0.02)	0.99	(0.01)	1.00	(0.00)
	90%	0.93	(0.08)	0.99	(0.01)	1.00	(0.00)	1.00*	(0.00*)	1.00*	(0.00*)	1.00*	(0.00*)

Data are mean and (SD) for five sets of 4D DIC microscope images of a *C. elegans *embryo from the one- to 24-cell stages.

*Data are for four sets of DIC microscope images, because no low-entropy regions were selected in one image set.

† Low-entropy regions were selected when they shared at least one pixel with regions already selected in the same focal plane at an adjacent time point, i.e., the same condition as used for the original forward and backward trackings.

‡ Low-entropy regions were selected when they shared at least one pixel with regions already selected in an adjacent focal plane at the same time point, i.e., the same condition as used for the original forward and backward trackings.

**Table 3:** Number of image sets for which advanced trackings detected nuclei with perfect sensitivity

		T_t_					
		
		1 pixel	4%	8%	12%	16%	20%
T_f_	1 pixel	5	5	5	4	4	4
	30%	5	5	4	4	4	3
	50%	5	5	4	4	2	1
	70%	5	5	4	4	2	1
	90%	1	1	1	1	0	0

Sensitivities of nuclear detection by low-entropy regions selected by advanced forward and backward trackings were calculated for five sets of 4D DIC microscope images of a *C. elegans *embryo from the one- to 24-cell stages. Number of image sets for which sensitivity of nuclear detection were 1.0 is shown.
