# Supplementary material for: Detection of nuclei in 4D Nomarski DIC microscope images of early Caenorhabditis elegans embryos using local image entropy and object tracking
Source: BMC Bioinformatics. 2005 May 24;6:125. doi: 10.1186/1471-2105-6-125 (PMC1175842; doi:10.1186/1471-2105-6-125)
Supplement: Additional File 1 — Detailed discussions about various measures of image texture. [file 1471-2105-6-125-S1.doc]

**Supplemental information**

“Detection of nuclei in 4D Nomarski DIC microscope images of early *Caenorhabditis elegans* embryos using local image entropy and object tracking”

Shugo Hamahashi, Shuichi Onami and Hiroaki Kitano

# **Investigation of the performance of widely-used texture measures in detection of nuclei in DIC images of early *C. elegans* embryos**

To select the texture measure used for quantifying the smoothness of image texture in our nuclear detection system, we investigated 25 widely-used texture measures selected from all four texture analysis methods categorized by Tuceryan and Jain [1]. The investigated texture measures are listed as follows:

1. Statistical methods
   1. first-order statistics

mean

standard deviation

skewness

kurtosis

energy

entropy

- 1. second-order statistics

co-occurrence: energy

co-occurrence: entropy

co-occurrence: contrast

co-occurrence: homogeneity

co-occurrence: correlation

autocorrelation

1. Geometrical methods

Voronoi tessellation features

Structural methods

1. Model based methods

Markov random fields

1. Signal processing methods
   1. Spatial domain filters

Laplacian operator

Kirsch operator

Prewitt operator

Moment M00

Moment M01

Moment M02

Moment M10

Moment M11

Moment M20

- 1. Fourier domain filtering

radial features of power spectrum

To evaluate the performance of these measures, we implemented them in accordance with methods given in the literature [1–3] and applied them to our 4D DIC microscope images of *C. elegans* embryos. We did not implement the Geometrical methods measures, because our DIC images were not composed of texture elements [1].

Of the resulting 23 measures, 11 measures discriminated between nucleus and cytoplasm in our DIC images of a *C. elegans* embryo (standard deviation; energy; entropy; co-occurrence: energy; co-occurrence: entropy; co-occurrence: homogeneity; co-occurrence: correlation; Markov random field; Kirsch operator; Prewitt operator; and Fourier domain filtering). We concluded that, among these 11 measures, entropy provided the best performance for our nuclear detection system, as follows.

Entropy was better than the four second-order statistics measures (co-occurrence: energy; co-occurrence: entropy; co-occurrence: homogeneity; co-occurrence: correlation) and the Fourier domain filtering measure in terms of computation time. The computation time was at least 6.5 times longer than that of entropy in the case of these second-order statistics measures, and more than 200 times longer in the case of the Fourier domain filtering measure. A shorter computation time is preferred in our system because our system was developed for detecting nuclei in 4D DIC microscope images of *C. elegans* embryos. A set of our 4D DIC microscope images usually consists of more than 6720 images from the one- to 24-cell stages.

Entropy is better than the two spatial domain filters (Kirsch operator and Prewitt operator) and the Markov random field in terms of sensitivity to differences in image quality. A detailed discussion on the spatial domain filters is given in the section “*Comparison of sensitivity to differences in image quality between the new system and our previous system*” in this Additional file. For the Markov random field, in more than 10 different DIC images of *C. elegans* embryos, we tried but could not find a set of parameters with which the measure successfully discriminated between nucleus and cytoplasm.

Entropy is better than the two first-order statistics measures (standard deviation and energy) because entropy discriminated between nucleus and cytoplasm more clearly than did these two measures. Standard deviation, like entropy, is a texture measure widely used to quantify the smoothness of image texture. In our test images, the standard deviation in the cytoplasmic region varied widely, and many small regions in the cytoplasm had standard deviations as small as in the nucleus. This wide variation in standard deviation in the cytoplasmic region may come from the dependency of standard deviation on actual pixel values in texture. Entropy does not depend on actual values in texture, but it does depend on the smoothness of texture. Energy discriminated between nucleus and cytoplasm far more clearly than did standard deviation. However, the difference in energy between the nucleus and cytoplasm was smaller than the difference in entropy.

# **Comparison of sensitivity to differences in image quality between the new system and our previous system**

The new nuclear detection system presented in this study is less sensitive than our previous system to differences in image quality [4] (Supplemental Figure S1). The difference in local image entropy (the image texture measure used in the new system) between nucleus and cytoplasm is remarkably greater than that in the image texture measure used in the previous system (hereafter called *Yasuda et al.’s measure*). The difference in local image entropy at the boundary between nucleus and cytoplasm is remarkably greater than that in Yasuda *et al*.’s measure.

Our previous system [4] used edge-detection operators, that is, Kirsch and the Prewitt operators, to detect nuclei from DIC images. Because these edge-detection operators are very sensitive to differences in the intensity of local pixels, the output values of these operators varied widely in both the nucleus and cytoplasm. Therefore, application of thresholding directly to the images resulting from image conversion using these operators did not effectively discriminate between nucleus and cytoplasm.

To deal with this problem, the previous system applied a blur filter to the images resulting from image conversion; this was Yasuda *et al*.’s measure. Because the blur filter reduced the variation of the output values of the edge-detection operators, application of thresholding to the resulting images discriminated between nucleus and cytoplasm. However, because of the limited difference between nucleus and cytoplasm and at the boundary between nucleus and cytoplasm (Supplemental Figure S1), nuclear detection using Yasuda *et al.*’s measure was very sensitive to differences in image quality. Therefore, the previous system required laborious hand-tuning of the thresholding each time a new image set was applied.

# **Supplemental Figure**


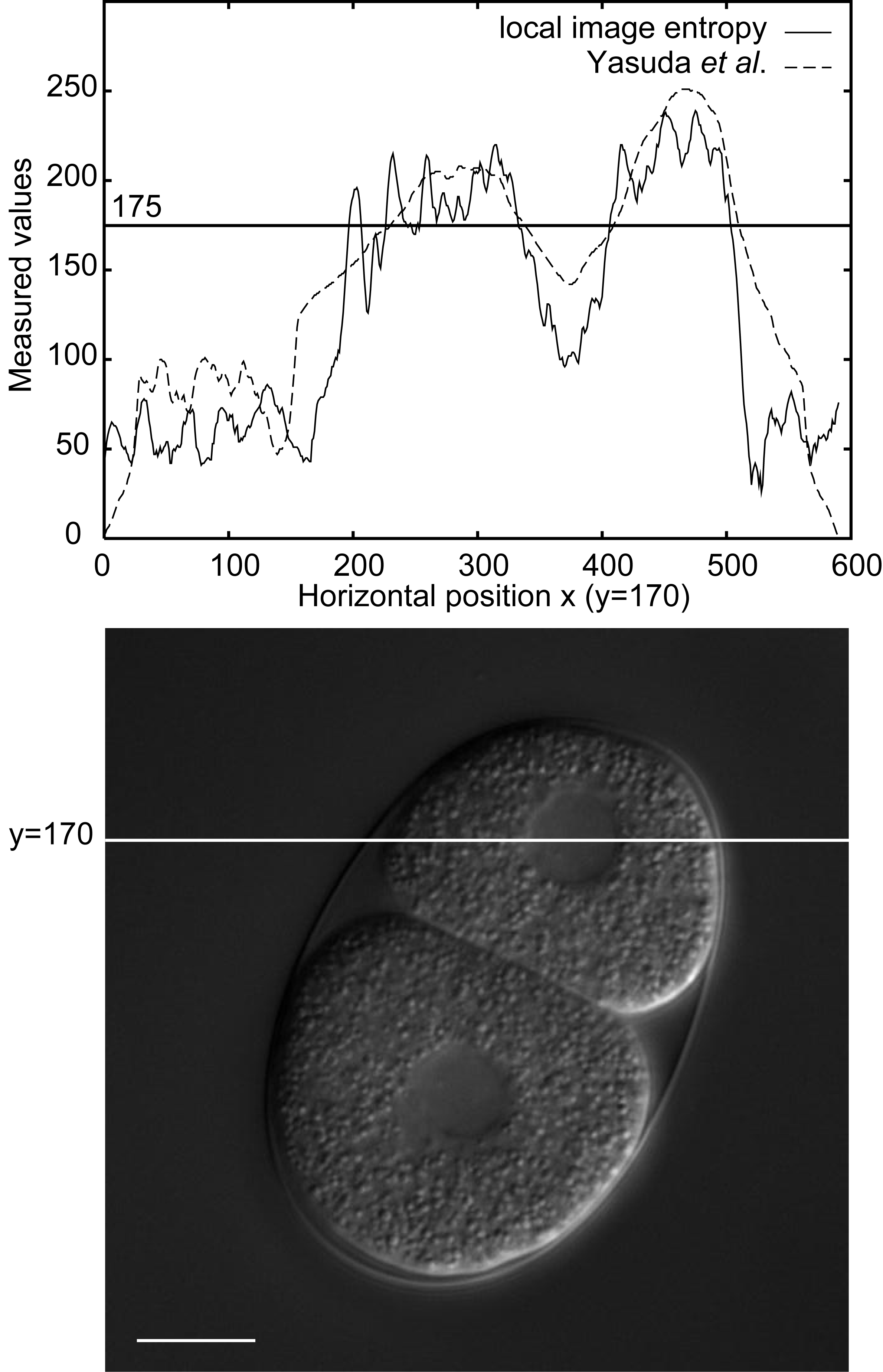


## **Supplemental Figure S1 – Values measured by local image entropy and Yasuda *et al.*’s measure**

The upper figure shows values measured by local image entropy and Yasuda *et al.*’s measure. The horizontal position in the upper figure corresponds to the horizontal position in the lower picture of y = 170. In the upper figure, the solid line shows the values of local image entropy and the broken line shows the values obtained by our earlier system (Yasuda *et al*.).

# References in the Supplemental information

1. Tuceryan M, Jain AK: **Texture Analysis**. In *The Handbook of Pattern Recognition and Computer Vision.* 2nd edition. Edited by Chen CH, Pau LF, Wang PSP. New Jersey: World Scientific Publishing Co.; 1998:207–248.
2. Pratt WK: *Digital Image Processing.* 2nd edition. New York: John Wiley & Sons; 1991.
3. Jähne B, Haußecker H, Geißler P (Eds): *Handbook of Computer Vision and Applications Volume 2: Signal Processing and Pattern Recognition*. San Diego: Academic Press; 1999.
4. Yasuda T, Bannai H, Onami S, Miyano S, Kitano H: **Towards automatic construction of cell-lineage of *C. elegans* from Nomarski DIC microscope images**. *Genome Inform* 1999, **10:**144–154.
