# Supplementary figures and images for: Detection of nuclei in 4D Nomarski DIC microscope images of early Caenorhabditis elegans embryos using local image entropy and object tracking
Source: BMC Bioinformatics. 2005 May 24;6:125. doi: 10.1186/1471-2105-6-125 (PMC1175842; doi:10.1186/1471-2105-6-125)

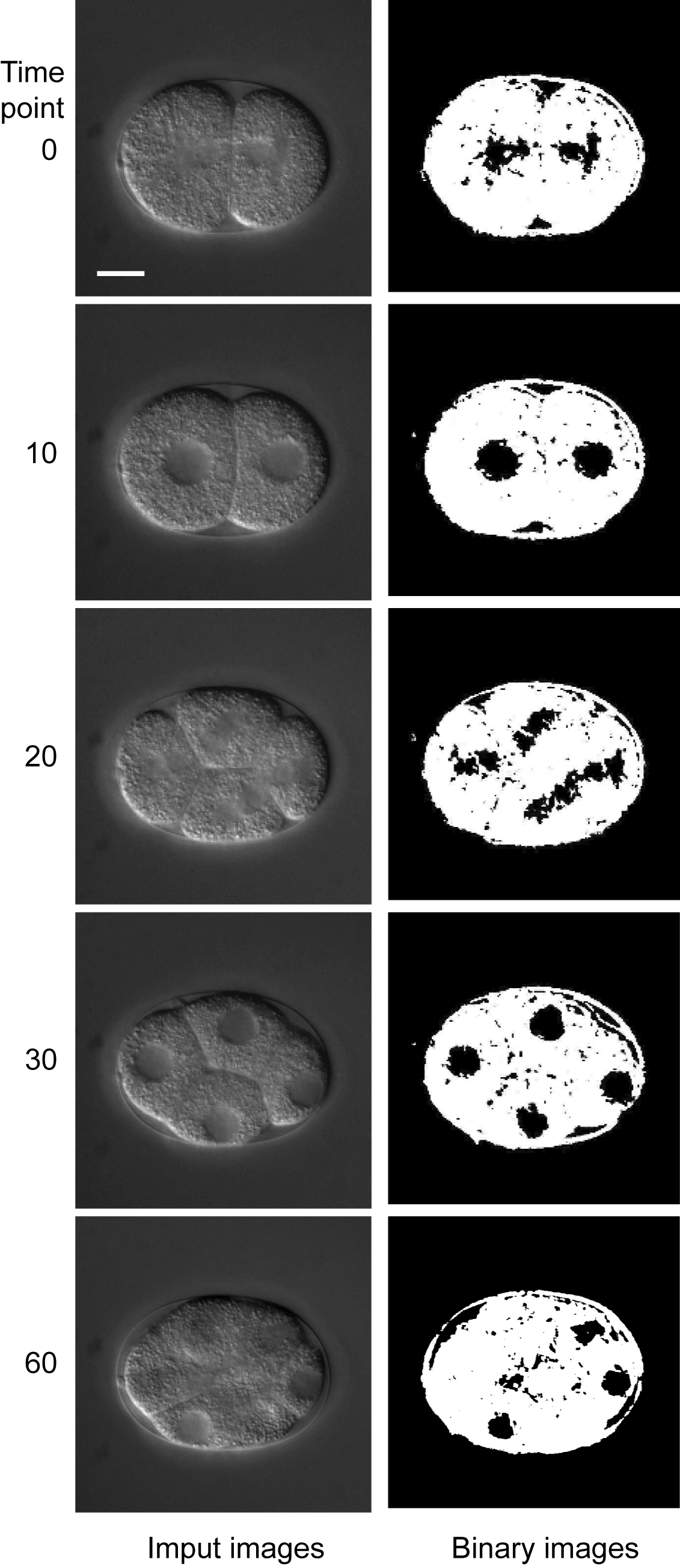

Supplement: Additional File 2 — Figure 5 Low-entropy regions in a par-1 embryo. Input images of a par-1 embryo are shown in the left column and low-entropy regions (black) are shown in the right column. Bar is 10 μm. [file 1471-2105-6-125-S2.tiff]

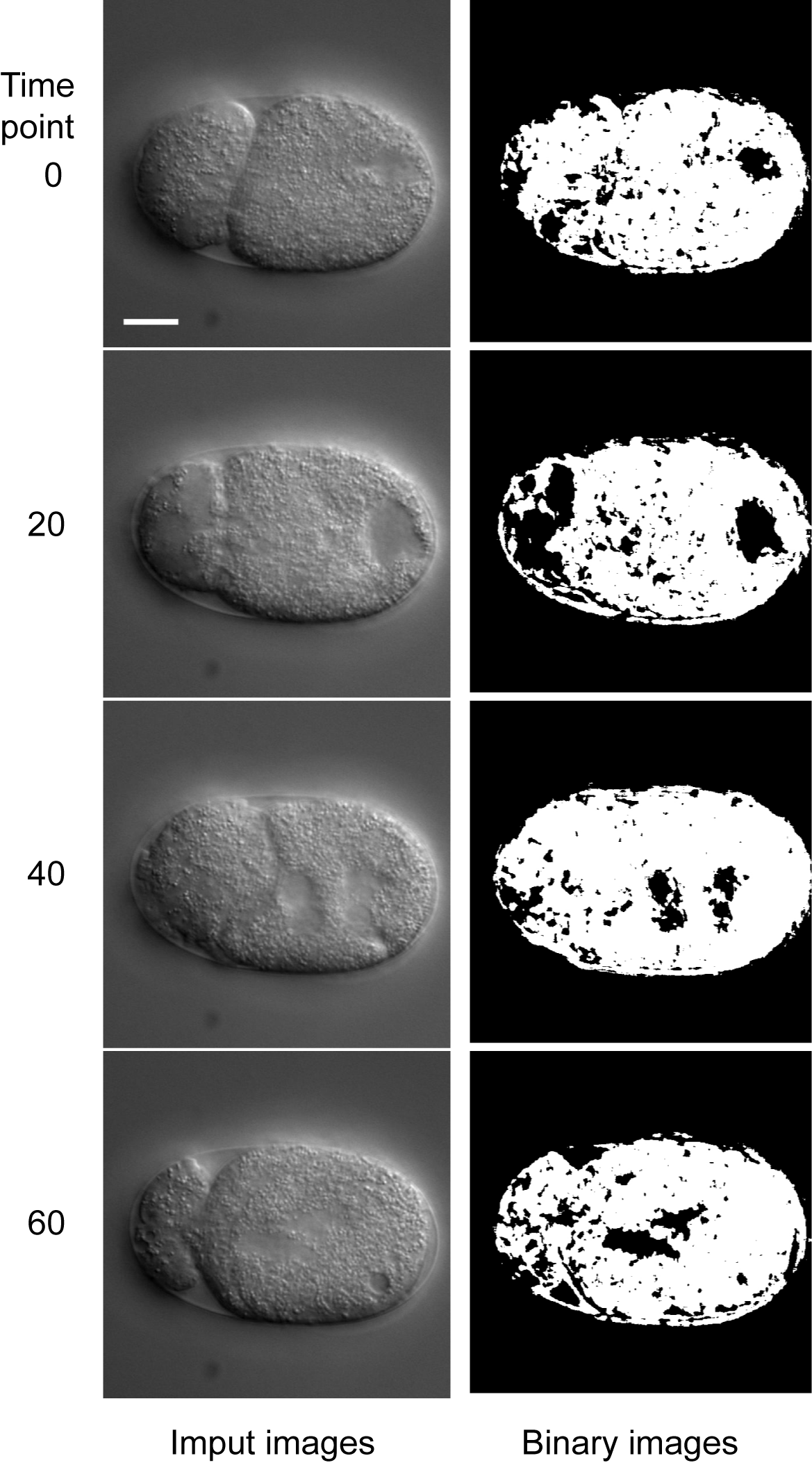

Supplement: Additional File 3 — Figure 6 Low-entropy regions in a tba-2 (RNAi) embryo. Input images are shown in the left column and low-entropy regions (black) are shown in the right column. Bar is 10 μm. [file 1471-2105-6-125-S3.tiff]
